# Microbiology of Peritonsillar Abscesses

**DOI:** 10.1016/S1808-8694(15)30063-X

**Published:** 2015-10-19

**Authors:** Flavio Akira Sakae, Rui Imamura, Luiz Ubirajara Sennes, Bernardo Cunha Araújo Filho, Domingos Hiroshi Tsuji

**Affiliations:** a MD, Postgraduate student of Otorhinolaryngology - University Hospital -USP.; b PhD in Otorhinolaryngology - USP, Assistant physician - University Hospital - USP.; c Associate Professor - University Hospital - USP, Associate Professor - University Hospital - USP.; d MD, Postgraduate student in Otorhinolaryngology - University Hospital - USP.; e Associate Professor - University Hospital - USP.

**Keywords:** Microbiology, Peritonsillar Abscess

## Abstract

**Aim:** The objective of the present study was to analyze the microbiology of peritonsillar abscesses. **Methods:** Thirty patients, mean age 24,2 years, with peritonsillar abscesses underwent aspiration of at least 3 mL of pus, which was cultured for aerobes and anaerobes. **Results:** 87% samples showed positive cultures. Aerobic or facultative aerobic bacteria were isolated from 23% aspirates, mixed aerobic and anaerobic bacteria from 60%, and anaerobic bacteria from only 3% aspirate. A total of 69 bacterial isolates (34 aerobic and 35 anaerobic) were recovered. The most common aerobic isolate was Streptococcus sp, with Streptococcus pyogenes being identified in 23% of aspirates. The predominant anaerobic isolates were Prevotella sp and Peptostreptococcus sp. Patients had received previous antimicrobial therapy in 63% cases. In this group, 1.8 isolates per specimen were recovered, a lower number than in the untreated group (3.0 per specimen). No significant difference in the species isolated was observed between these two groups. **Conclusion:** Peritonsillar abscess is usually a polymicrobial infection, with predominance of anaerobic bacteria. The number of agents isolated was larger in patients not previously treated with antibiotics, but the use of antimicrobial drugs did not interfere with the type of bacterium isolated.

## INTRODUCTION

Peritonsillar abscess is characterized by a purulent secretion collected between the fibrous capsule of the palatine tonsil and the pharyngeal superior constrictor muscle^1^, ^2^. The proper treatment for this infection aims at avoiding serious complications which include its extension into deep spaces in the neck and its rupture with secretion being aspirated towards lower airways. Its management varies in the literature, from pus aspiration and/or drainage all the way to tonsillectomy^2^, ^3^.

Antibiotics play a fundamental role in its treatment; however the first choice drug to be used is still a very controversial topic. Penicillin is considered a first choice drug because of their action on Streptococcus pyogenes - a germ that has been traditionally associated to this infection1. Notwithstanding, with the progress we have seen in microbiological culture techniques, anaerobic germs were isolated and are now considered important in this disease. The goal of this present study is to assess the microbiology of these abscesses seen in the ENT emergency room of our facility.

## MATERIALS AND METHODS

This study has been approved by the Ethics Committee for Research Project Analysis - CAPPesq - Clinical Board - University Hospital - University of São Paulo.

We carried out a prospective study with 30 patients diagnosed with peritonsillar abscess at the ENT ER of the University Hospital of the University of São Paulo, from June to November of 2001. Patients’ ages varied between 9 and 69 years, with an average of 24.2 years. Most of them were females (66.7%). Data about prior use of antibiotics were obtained through history taken at the ER.

All patients underwent puncture with sterile #14 Gelco® in the peritonsillar region of the largest bulging, with aspiration of purulent secretion (more than 3ml of pus). Pus was confirmed, and then the mucosa was incised and dissected with a Kelly-type forceps, to drain the pus collection. Thirty aspirates were included in the study.

After proper puncture, the material was immediately placed in a sterile, dry and vacuum tube and referred to the microbiology lab within 30 minutes. There, the material was then separated in order to carry out aerobic and anaerobic cultures. The aerobic cultures were placed in blood agar, chocolate agar and MacConkey dishes, incubated under a 4% CO2 atmosphere, at 37°C for 48 hours. Anaerobic cultures were incubated in anaerobic atmosphere dishes, at 37°C for 3 to 7 days. The germs were identified in the conventional way.

The aspirates were analyzed as to culture positiveness index, total number of bacteria found by aspirate, and separated as to the use or not of antibiotics prior to aspiration. We also studied the ratio of anaerobic and aerobic germs found, specifying each etiological agent found.

We separated the microorganisms in three major groups: Streptococcus sp, other aerobic and anaerobic agents and we compared their isolate frequencies according to the use or not of antibiotics prior to puncture.

## RESULTS

In 19 (63%) episodes of peritonsillar abscess, the patients had used antibiotics before coming to our ENT ER service, and all of them had used penicillin derivatives.

Of the 30 aspirates, in 26 at least one microorganism grew, resulting in a positiveness index of 86.7% in the cultures. In 7 (23.3%) aspirates only aerobic or facultative bacteria grew, in 1 (3.3%) only anaerobic bacteria grew, and finally, in 18 (60%) aspirates, both aerobic and anaerobic bacteria grew.

A single bacterium was found in 3 (10%) aspirates, with Streptococcus pyogenes growing in two aspirates and Veillonella parvula in one. In the other 23 (76.7%) aspirates more than one bacterium grew.

A total of 69 bacteria were found, in average this corresponded to 2.3 bacteria per aspirate (1.1 aerobes and 1.2 anaerobes). In patients that used antibiotics prior to puncture we found 1.8 bacteria per aspirate (0.7 aerobes and 1.1 anaerobes) and among those who did not use we found 3 bacteria per aspirate (1.7 aerobes and 1.3 anaerobes). In the 4 aspirates with negative cultures, the patients had used antibiotics prior to the puncture.

**Chart 1:**
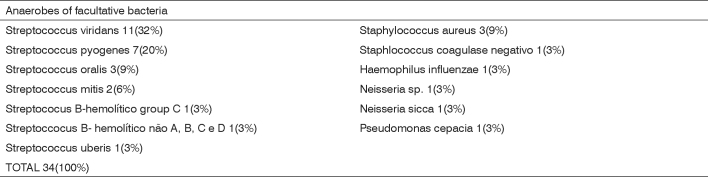
Anaerobe or facultative bacteria identified in 30 peritonsillar abscesses.

**Chart 2:**
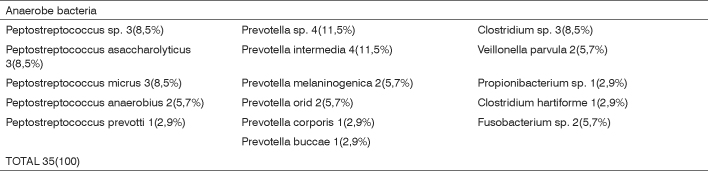
Anaerobe bacteria identified in 30 peritonsillar abscesses.

We found 34 aerobic or facultative bacteria (Chart 1). Prevailing aerobes were Streptococcus sp (26 isolates, including 7 Streptococcus pyogenes) and Staphylococcus sp (4 isolates, 3 Staphylococcus aureus). Chart 2 shows that we isolated 35 anaerobic bacteria. The predominant anaerobes were Prevotella sp (14 isolates) and Peptostreptococcus sp (12 isolates).

**Chart 3:**
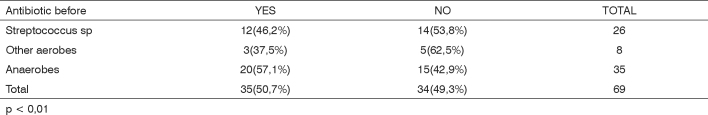
Microorganisms divided according to the use or not of antimicrobial agents before puncture.

There were no statistical differences on the type of bacteria isolated according to the groups defined (Streptococcus sp, other aerobes and anaerobes) as to the use or not of antibiotics prior to puncture (Chart 3).

## DISCUSSION

In our study, the culture positiveness index was 86.7%, similar to what was found by Mitchelmore^1^ (85%) and Jokipii^4^ (88.1%). This high positiveness index suggests that the methodology used was adequate to favor bacterial growth, including those bacteria which are difficult to isolate, such as anaerobes. Some factors, such as the collected volume of more than 3ml, the use of vacuum tubes and the fast transportation to the lab may have favored the growth of these microorganisms.

Streptococcus pyogenes, a common bacterium found in acute tonsillitis, was considered important in peritonsillar abscesses because of the supposed relationship between both diseases^4^. Notwithstanding, in most of the abscess cases in this study, such agent was not present, it occurred in only 7 (23.3%) samples. This result was similar to that found by Snow5 (23%), Flodstrom6 (24%) and Jokipii^4^ (23.8%).

On the other hand, anaerobic organisms were found alone or together with other aerobic or facultative organisms present in most of our patients (63.3% of the aspirates), thus confirming the importance of these agents in peritonsillar abscesses. Some authors have described even higher isolate frequencies for anaerobes, ranging from 66.7% to 94%^1^, ^4^, ^7^.

These microbiologic findings may suggest that peritonsillar abscesses and acute tonsillitis represent different outcomes of the same disease, or that the abscess pathogenesis would have no relationship with acute tonsillitis, but rather with other infections such as Weber peritonsillar glands infections, as suggested by Passy^8^.

Most of the peritonsillar abscess cases have a polymicrobial infection^4^, ^9^. In our study we found 2.3 bacteria per aspirate, however other authors found even larger figures, such as Jokipii^4^ (3.2 organisms per sample), Jousimies-Somer^10^ (4.4) and Brook^7^ (3.1).

As far as the etiological agents for peritonsillar abscesses are concerned, the most commonly found aerobic bacteria was Streptococcus sp, and this finding is in agreement to what has been reported in the literature. While among anaerobes, Fusobacterium sp and Peptostreptococcus sp were the ones most found^4^, ^10^, ^11^, ^12^, in our study, the most frequently found were Prevotella sp and Peptostreptococcus sp. Brooks et al.^7^ found Bacteroids sp as the most frequent anaerobe present in the aspirates. In our study we found no Bacteroids, which are an agent present in the normal oral cavity and oropharynx flora, and its presence in the culture may represent sample contamination.

The variations found in the results of cultures from different studies, both in anaerobe detection frequence as well as in number of microorganisms present per aspirate may be explained by certain factors. First, according to Hall^13^, results differ according to geographic locations and microbiology technique used. Besides, we know that the tonsil surface is covered by the bacteria present in the normal flora, and the most common are Streptococcus, Neisseria from aerobes, and Peptostreptococcus, Veilonella, Actinomyces and Fusobacterium from anaerobes, in the ratio of 1 aerobe for 100 anaerobes^14^. During aspiration, there is the possibility of contamination by organisms from the normal flora, therefore, culture results should be interpreted carefully when these organisms are found (aerobes and anaerobes). And finally, for anaerobic organism cultures, it is essential to have proper aspirate transportation and processing, emphasizing the medium used and the incubation period, for a successful identification of microorganisms responsible^1^.

In this study, most of the bacteria (3 per aspirate) were found in patients who did not use antibiotics prior to puncture, however without significant difference among the organisms found when compared to the patients who used antibiotics before the puncture.

Peritonsillar abscess treatment is still controversial. Antibiotic agents are used empirically, even before any culture has been obtained^4^. Usually, if the patient is clinically improving, the antibiotic agent is not changed in fear of altering treatment response. In his study, Cherukuri et al.^15^, concluded that the treatment of peritonsillar abscess does not require culture because no treatment has been modified based on culture results. Although 74% of the patients used clindamycin as initial treatment.

The choice of drug to be used in cases of peritonsillar abscess depend on the related etiological agents^7^, our study leads us to believe that the best treatment for this infection is to use broad spectrum antibiotics against aerobic and anaerobic agents. Besides, immediate incision and drainage is the treatment of choice, because severe complications accruing from peritonsillar abscesses are seen in emergency rooms.

## CONCLUSION

In most of cases, peritonsillar abscesses present polymicrobial infections, and the most important agents are the anaerobes. S. pyogenes, a germ frequently associated to these infections, was isolated in only 23.3% of samples. The number of germs found is greater in patients who did not use antibiotics prior to drainage; however the use of antimicrobial agents did not interfere with the type of bacteria identified.
